# Concluding Remarks for Special Issue HTLV-HIV Co-Infections

**DOI:** 10.3390/v15040963

**Published:** 2023-04-14

**Authors:** Alejandro Vallejo, María Abad-Fernández

**Affiliations:** 1 Laboratory of Immunovirology, Infectious Diseases Department, Health Research Institute Ramon y Cajal (IRyCIS), Ramon y Cajal University Hospital, 28034 Madrid, Spain; 2 UNC HIV Cure Center, University of North Carolina, Chapel Hill, NC 27599, USA

During the early 1980s, the first 3 human retroviruses were identified: human T-lymphotropic virus 1 and 2 (HTLV-1 and HTLV-2) and human immunodeficiency virus (HIV). A person living with HIV has a higher probability of acquiring HTLV-1 or HTLV-2 infection than the general population, as they share mainly the same routes of transmission. Hence, HTLV-HIV co-infections are frequently detected in vulnerable populations, such as intravenous drug users (IDU). Furthermore, IDUs also have an increased risk of acquiring an HCV infection. In general, HIV-1 and HTLV-1 co-infection is associated with shorter survival, a higher mortality rate, and faster progression to death, while co-infection with HIV-1 and HTLV-2 seems to have an association with longer survival, slower AIDS progression, and a lower mortality rate. On the other hand, HCV/HTLV-1 co-infection has been associated with the worse outcome of HCV infection worldwide (higher HCV viremia, lower rate of sustained virological response to α-interferon treatment, increased risk of chronic liver disease and cancer). This Special Issue contains three original studies and one review that contribute to the overall knowledge of HTLV-HIV coinfection.

From the first original study, we learn that CD8+ T cell-mediated HIV-1 inhibition in vitro was higher in individuals with HTLV-2 infection. This inhibition activity was associated with a higher frequency of effector memory CD8+ T cells, higher levels of granzyme A and granzyme B cytolytic enzymes, and perforin. Hence, cellular and soluble cytolytic factors may contribute to the lower HIV-1 pre-antiretroviral treatment viral load. Also, HIV-1 proviral load is associated with HTLV-2 infection. The authors confirmed and expanded previous findings on the role of HTLV-2 in the beneficial effect on the pathogenesis of HIV-1 in coinfected individuals [1].

In the second study, authors measured cytokines/chemokines, CD4 and CD8 T cells, and HIV viral load (VL) in HIV single-infected and co-infected individuals matched for age and sex and divided into six groups (G1 (69 HIV); G2 (9 HIV/HTLV-1); G3 (6 HIV/HTLV-2); G4 (11 HIV/HCV); G5 (19 HIV/HCV/HTLV-1); and G6 (15 HIV/HCV/HTLV-2)). They reported that the highest levels of Th1 and pro-inflammatory cytokines were detected in G2 (IFN-γ) and G6 (IL-6 and IL1-β), and of chemokines in G1 (MIG, IP10, RANTES), G4 (MCP1), and G6 (MIP1-β). Additionally, the highest CD4 cell number and the lowest HIV VL were identified in G3, and the opposite results in G2. They found positive correlations between CD4 and CD8 cell counts, and IL-6 levels were detected in G2 and G5, and of HIV VL and RANTES in G4. They also found negative correlations between CD8 and IFN-γ in G4 and HIV VL and RANTES in G6. The authors suggest a negative impact of HTLV-1 and a possible protective role of HTLV-2 in HIV infection progression in such patients [2].

The third study analyzes the use of mathematical modeling to support biological and medical research on within-host HIV-1/HTLV-I co-infection dynamics. The effects of humoral immunity and C-T-C transmission on the HIV-1/HTLV-I co-infection dynamics are discussed. The authors have shown that humoral immunity does not play the role of clearing an HIV-1 infection, but it can control HIV-1 infection. Furthermore, the omission of C-T-C transmission from the HIV-1/HTLV-I co-infection model leads to an under-evaluation of the basic HIV-1 mono-infection reproductive ratio [3].

The review contains current knowledge on dendritic cell (DC) sensing of HIV-1 and HTLV-1, including DC-SIGN, Tat, and Tax, as well as current viral therapies. An overview of DC interaction with oncogenic viruses, including EBV, Hepatitis viruses, and HPV, is also provided. Vaccines and therapeutics targeting host–pathogen interactions can provide a solution to co-infections, neurodegeneration, and cancer [4].

We would like to acknowledge all authors for their contributions to this HTLV-HIV Coinfection Special Issue. It has been a pleasure to read and learn. Together, we will continue our research to understand these co-infections, including the mechanisms that determine infection, transmission, and pathogenesis.
